# Mixed Epithelial and Stromal Tumor of the Kidney: Clinicopathologic Features and Surgical Outcomes in Four Patients

**DOI:** 10.7759/cureus.77562

**Published:** 2025-01-16

**Authors:** Yateesh H M, Sadiq Nawaz Fayaz, Sri Ram Ganesh, N Muhammed Lazim, Rajeev TP, Rana Fathima

**Affiliations:** 1 Surgery, Yenepoya Medical College Hospital, Mangalore, IND; 2 Urology, K S Hegde Medical Academy, Mangalore, IND

**Keywords:** adult cystic nephroma (acn), clinicopathologic features, kidney neoplasms, mixed epithelial and stromal tumor (mest), surgical resection

## Abstract

Mixed epithelial and stromal tumor (MEST) is a rare neoplasm of the kidney that is seen mostly in middle-aged women with a history of hormonal contraceptive consumption. Mixed epithelial and stromal tumors are usually benign tumors with minimal chances of metastasis and recurrence. However, few malignant transformations of MEST have been recorded. In perimenopausal patients presenting with a complex cystic renal mass in the renal system with a history of selective estrogen receptor modulator (SERM) therapy, MEST must be a differential diagnosis. The key treatment in MEST is surgical resection of the tumor. A MEST is similar to adult cystic nephroma (ACN), but MEST has complex epithelial and stromal components.

We present a case series of four patients with a characteristic tumor of the kidney with the presence of mixed epithelial and stromal components. The ages of the patients ranged from 48 years to 55 years (mean age being 51 years). One of these cases is relatively rare as the patient is male and has no history of taking hormonal medications. Patients underwent surgical removal of tumors, and all of the patients recovered well postoperatively.

## Introduction

A rare renal neoplasm primarily seen in middle-aged women known as a composite epithelial and stromal kidney tumor is often associated with a history of hormonal therapy, including contraceptive use. They tend to extrude through the pelvicalyceal system. Extension into the pelvis, upper ureter, and vesicoureteric junction has been reported [1, 2]. About 100 cases have been described in the literature [3]. Here we are reporting four incidental cases of mixed epithelial and stromal tumor (MEST); one among these unique being arising from the right renal pelvis running through the entire right ureter extruding into the bladder from the right ureteric orifice, and another in a male individual with no prior medical background of any antiestrogenic medications. All four of the patients presented with symptoms of pain in the loin. One patient also complained of abnormal uterine bleeding. All the women reported having hormonal therapy. Grossly, the tumors were well-circumscribed and had solid and cystic components, with gelatinous material on the cut section. Histopathological examination revealed a biphasic pattern consisting of columnar epithelial cells and spindle-shaped stromal components.

## Case presentation

Case one

A 52-year-old perimenopausal female exhibited the dull, aching type of pain in the right loin associated with menorrhagia exacerbated for five months. Being diagnosed as a case of abnormal uterine bleeding (AUB). She had a history of taking ormeloxifen, a selective estrogen receptor modulator (SERM), for a duration of one year. Clinical examination revealed tenderness in the right lumbar region. She was anemic with a hemoglobin of 9.6 g/dl and hematocrit of 30.8%. An ultrasonogram of the abdomen revealed a frond-like echogenic lesion of 36x30mm in the vicinity of the bladder trigone on the right side with a dilated right ureter and right renal pelvis. The uterus was bulky with significant endometrial collection, and loss of endometrial junctional planes was noted.

**Figure 1:**
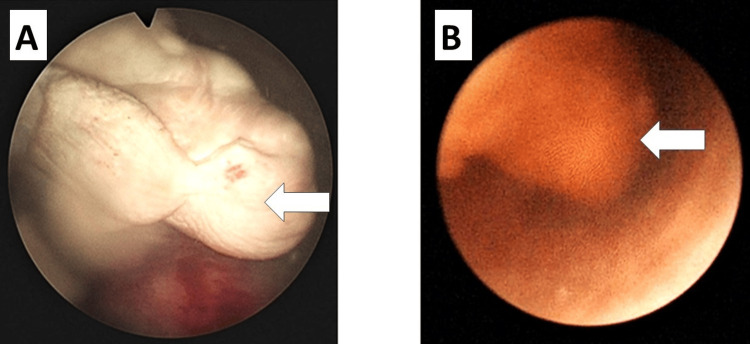
(A) Cystourethroscopy image showing a tumor in the right renal pelvis; (B) Cystoscopy image showing a growth extruding into the bladder

**Figure 2:**
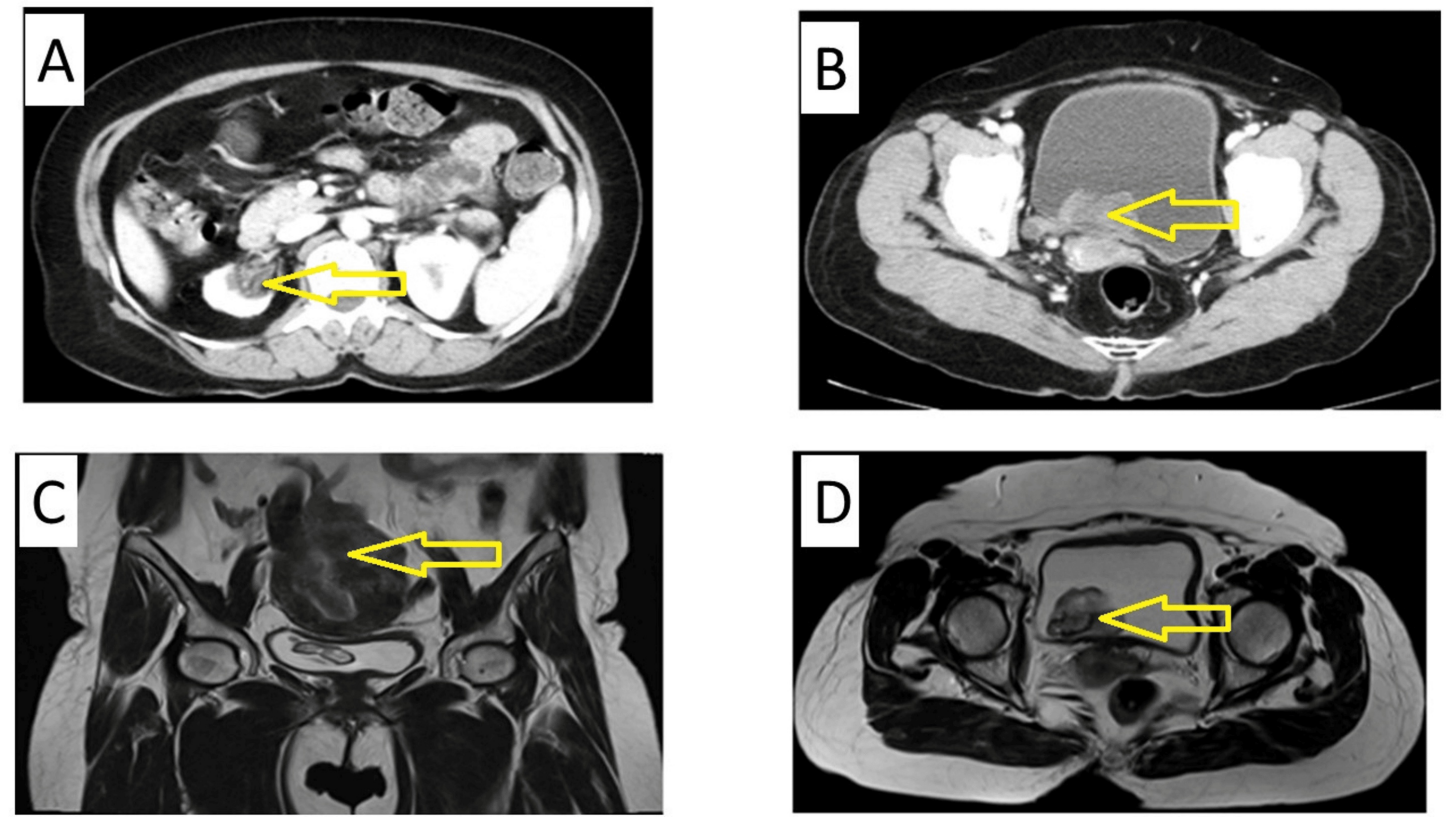
A CT image showing a tumor arising from the right renal pelvis running through the entire right ureter extruding into the bladder (A) from the right ureteric orifice (B) and MRI images showing the tumor extruding into the bladder lumen (C) and (D). CT: computed tomography; MRI: magnetic resonance imaging

Diagnostic cystourethroscopy (Figure 1) showed a tumor arising from the right renal pelvis running through the entire right ureter, extruding into the bladder from the right ureteric orifice. Contrast-enhanced computed tomography (CT) with a urographic CT scan and magnetic resonance imaging (MRI) of the abdomen and pelvis was performed. Endophytic pedunculated enhancing mass lesion, measuring 2.8x3.7x1.8 cm, arising from the right vesicoureteric junction, extending into the right distal ureter, causing dilatation of the ureter, for a length of about 15 cm was observed (Figure 2). A small contracted right kidney having Grade IV hydroureteronephrosis with an indistinct fat plane between the inferior vena cava (IVC) and uterus was noted. The patient was diagnosed with AUB with suspicion of urothelial carcinoma of the right pelvis.

**Figure 3:**
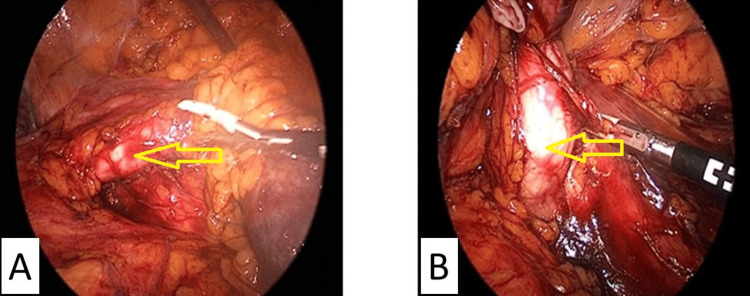
Intraoperative images showing a dilated ureter and right kidney Both the images are intraoperative and the tumors have been marked by an arrow.

**Figure 4:**
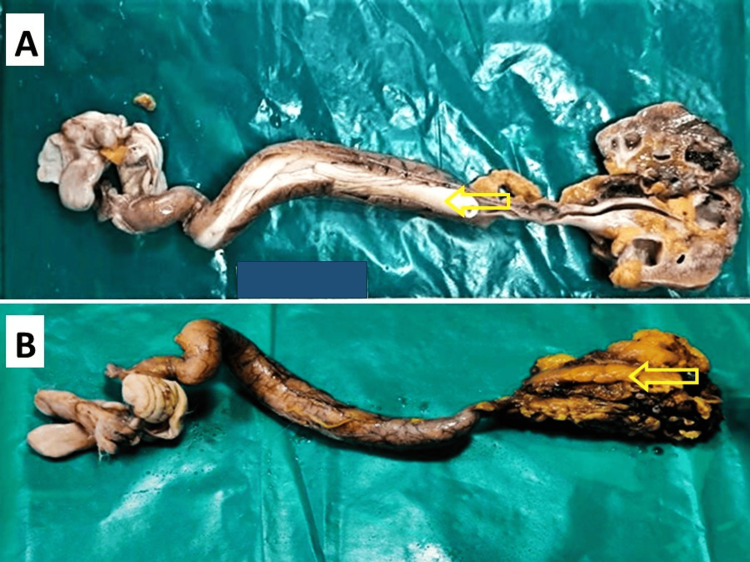
(A) Pale white polypoidal growth from the distal pelvicalcyeal system and proximal ureter renal pelvis containing fibrofatty tissue containing gelatinous fluid; (B) Shrunken right kidney with congested outer surface covered with perinephric fat, dilated ureter measuring 21cm with endophytic polypoidal growth, extending into the bladder

She underwent laparoscopic right radial nephroureterectomy and bladder cuff excision (Figure 3) combined with bilateral salpingo-oophorectomy and hysterectomy. On gross examination (Figure 4), the shrunken right kidney measured 6.3x4.6x2.7 cm, with the ureter measuring 21 cm. The outer surface of the kidney was congested and had perinephric fat. The outer surface of the ureter exhibited greater dilation in the middle and distal portions. Pale white polypoidal growth from the distal pelvicalyceal system and proximal ureter with renal pelvis having fibrofatty tissue containing gelatinous fluid was noted. The ureter had pale white, cylindrical endophytic growth measuring 16 cm in length. Pale white polypoidal growth is seen on the outer surface in continuation with the cut surface of polypoidal growth on the outer surface.

**Figure 5:**
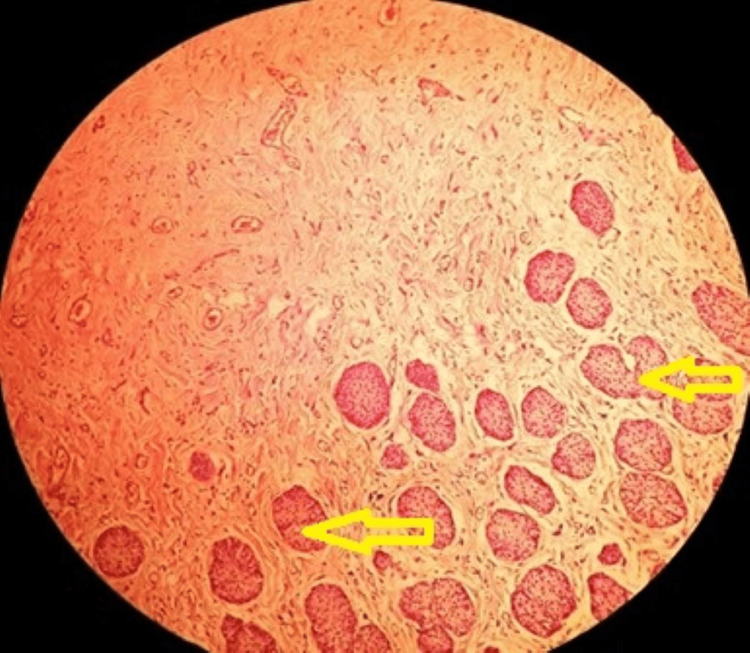
Urothelium arranged in nests and focal tubules Stroma appears loose and fibromyxoid as shown in the histopathological slide.

Microscopic analysis (Figure 5) revealed a two-phase pattern with epithelial elements comprised of columnar cells and stromal components of spindle cells with abundant cytoplasm with plump nuclei. A fibromyxoid stroma with thick cystic septae extending to form solid areas of the tumor was observed. The urothelium at places had been arranged in nests and focal tubules. With these findings, a diagnosis of MEST was made. The postoperative period was uneventful, and the patient recovered well.

Urine cytology was not performed, and no biopsy was taken during cystoureteroscopy as the imaging findings and clinical presentation strongly suggested a malignant lesion, prompting definitive surgical management. Intraoperatively, no significant lymphadenopathy was observed; therefore, lymphadenectomy was not performed. Immunohistochemistry (IHC) confirmed a biphasic pattern of epithelial and stromal components, consistent with a diagnosis of MEST.

Case two

A 50-year-old post-menopausal woman presented with dull, aching pain in her left flank for three months. She presented with a left flank mass measuring 15 cm × 10 cm, palpable upon bimanual examination. Blood tests were within normal limits. Urinalysis showed the presence of microscopic blood in the urine, and urine cytology detected abnormal cells. A contrast-enhanced CT scan revealed a complicated, multi-cystic lesion within the left kidney that extended into the pelvicalyceal system and reached the left vesicoureteric junction (VUJ). Cystoscopy showed no abnormalities. A retrograde ureterogram revealed that the ureteral catheter could not be advanced past the VUJ. Suspecting a transitional cell carcinoma in the upper left tract, the patient was scheduled for a left nephroureterectomy. During the procedure, a sizable kidney tumor was identified. The full length of the left ureter, extending to the VUJ, was markedly expanded, twisted, and contained a palpable lobulated mass. Consequently, a left nephroureterectomy was performed, including the removal of the VUJ and a portion of the adjacent bladder cuff. The postoperative period and course to date have been uneventful.

**Figure 6:**
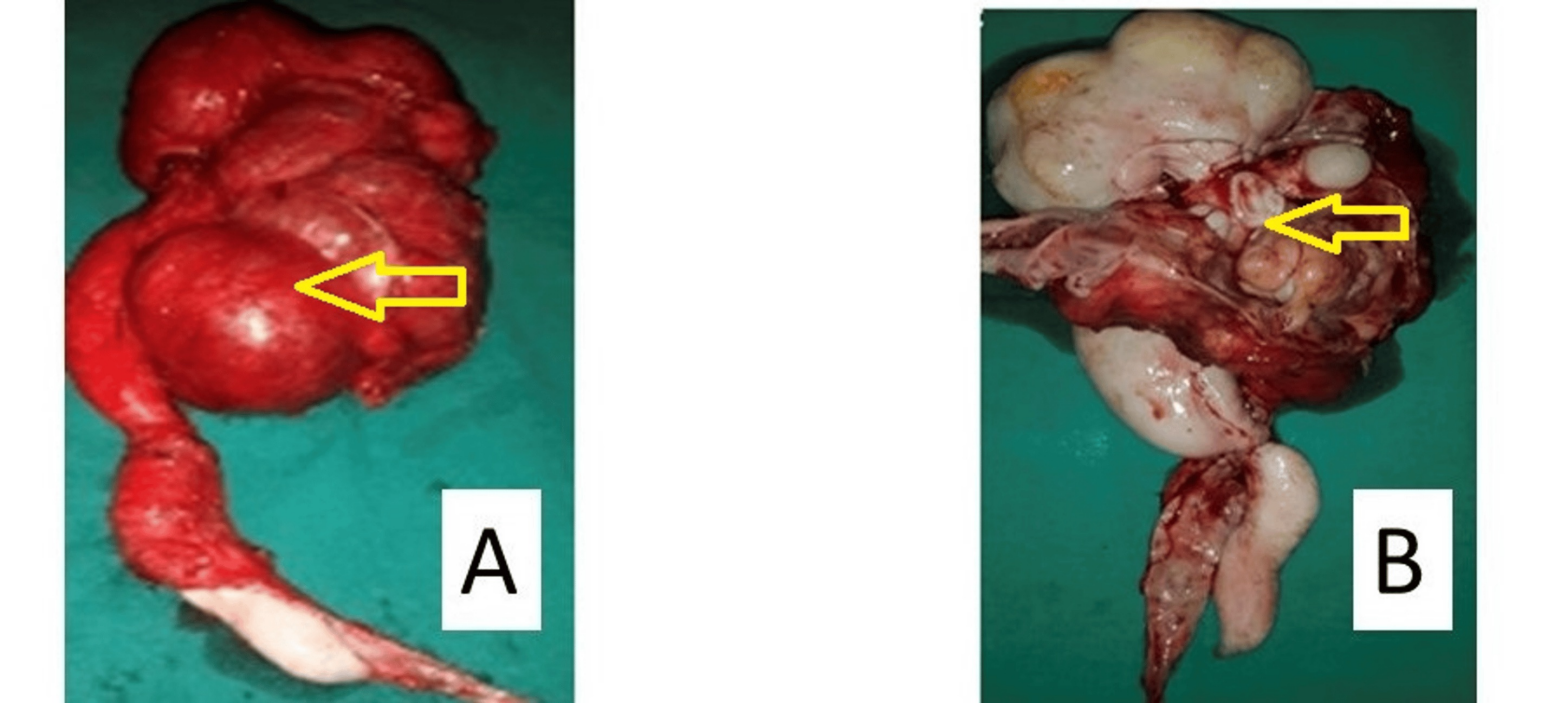
(A) Left kidney with a bosselated surface; (B) A pale, white, polypoid mass originating in the central region of the kidney and spreading into the pelvicalyceal system as well as the ureter.

A gross pathological examination (Figure 6) revealed a markedly enlarged left kidney with a lobulated surface. A pale, white, polypoid growth was noted originating from the central area of the kidney, extending into the pelvicalyceal system and the ureter up to the VUJ. The cut surface of the tumor displayed a gelatinous appearance. 

Urine cytology detected abnormal cells, raising suspicion of malignancy; however, no biopsy was taken during cystoureteroscopy to avoid potential tumor spillage and due to the high-risk nature of the lesion on imaging. Intraoperatively, no significant lymphadenopathy was observed, and lymphadenectomy was not performed. The IHC staining confirmed the lesion as transitional cell carcinoma, with positive staining for GATA3, CK7, and CK20.

Case three

**Figure 7:**
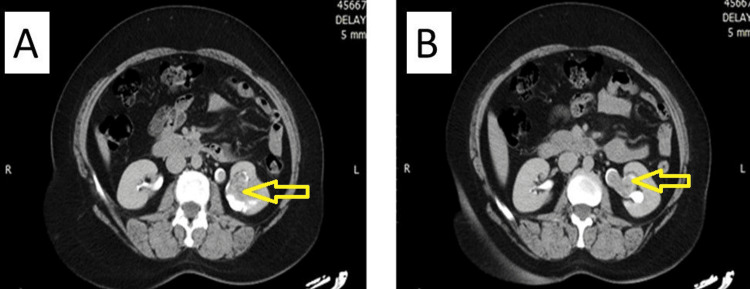
(A) A CT image showing a tumor arising from the lower pole of left kidney; (B) Extension of the tumor into the left renal pelvis CT: computed tomography

A 48-year-old perimenopausal female presented with dull, aching pain in the left loin, burning micturition for two months, and one episode of hematuria with clots in the urine. She was diagnosed with hypothyroidism and was on thyroxine hormonal supplementations. She had a history of taking oral contraceptives. Clinical examination revealed tenderness in the left lumbar region. An ultrasonogram of the abdomen revealed a 3.4x3.6 cm left kidney lower pole mixed echogenicity exophytic lesion with a predominant solid component. On cystourethroscopy, 4x4 cm of mass protruding from the inferior calyx into the renal pelvis was noted. Venous contrast CT and urographic CT scan of the abdomen were performed (Figure 7). A large, well-circumscribed, heterogeneously enhancing exophytic soft tissue attenuation mass arising from the posterior cortex of the lower pole region of the left kidney with extension into the lower calyx and left renal pelvis, confined within the renal capsule 3.4x3.6 cm. She was suspected to have urothelial carcinoma of the left renal pelvis.

**Figure 8:**
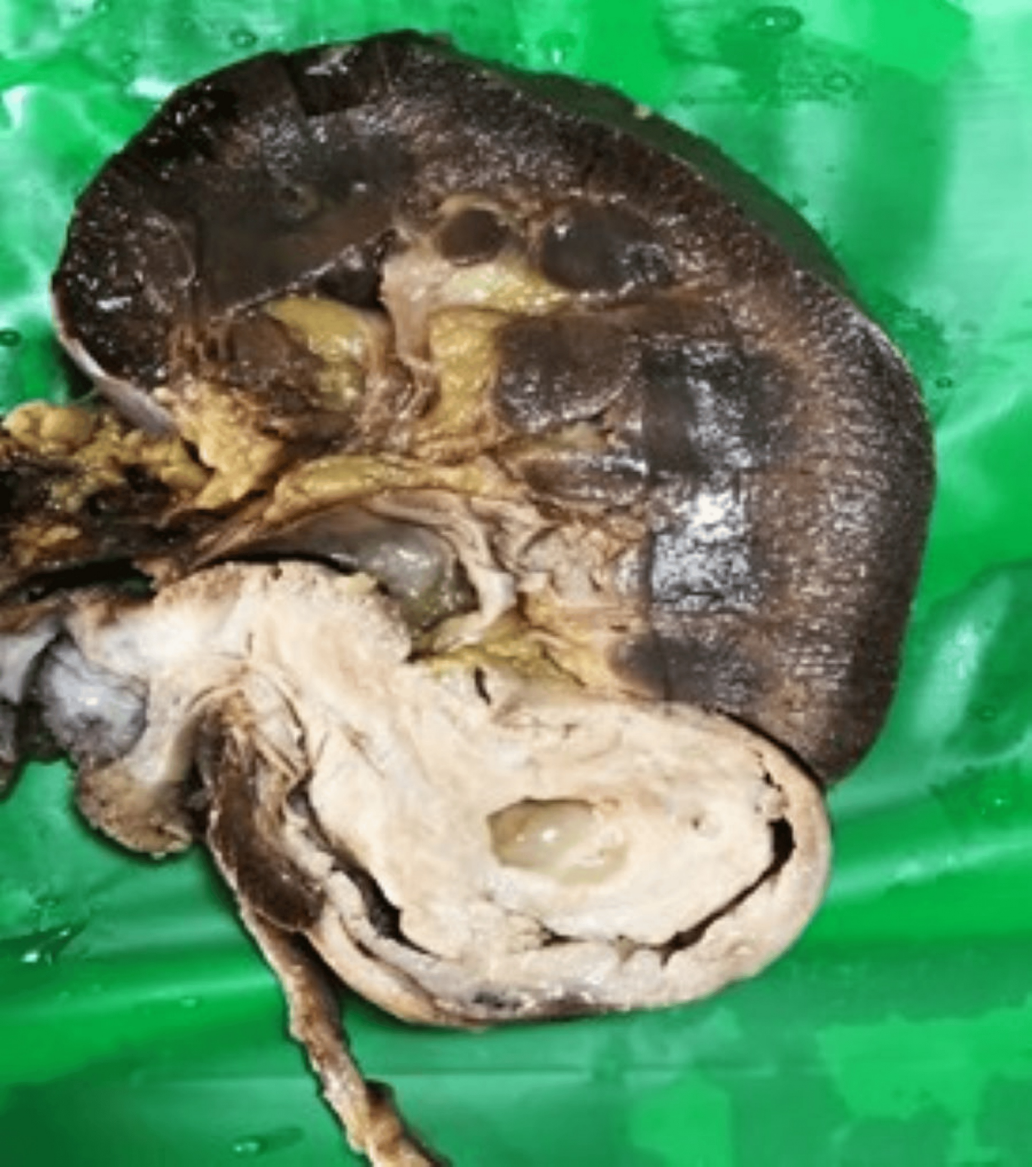
A greyish white mass bulging into the pelvis and the proximal portion of ureter

**Figure 9:**
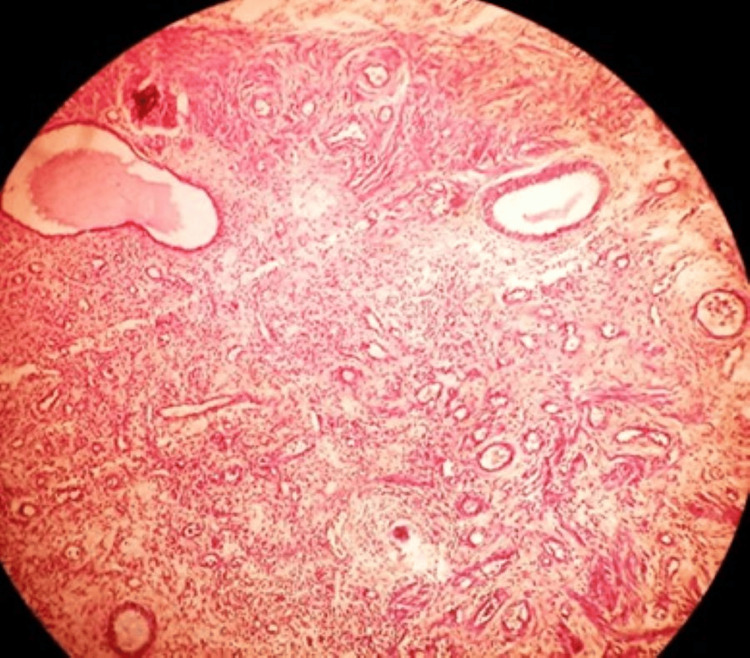
A well-circumscribed tumor comprising cysts and glands of differing sizes, lined with cuboidal urothelial cells and fibromyxoid stroma with cystic septae forming solid areas of the tumor

The patient underwent a left laparoscopic radical nephroureterectomy. Gross pathologic examination showed a well-demarcated gray-white lesion (Figure 8) measuring 8x3.7x2.7 cm in the lower pole, having both solid and cystic areas, bulging into the proximal portion of the ureter into the hilum and breaching the capsule to produce a protrusion on the outer aspect involving the cortex and medulla. Microscopy (Figure 9) showed well-circumscribed tumor areas comprising cysts and glands of differing sizes, lined with cuboidal urothelial cells and fibromyxoid stroma with cystic septae forming solid areas of the tumor. The postoperative period was uneventful, and the patient has been doing well in the last 12 months with no complaints.

A biopsy was not performed during cystoureteroscopy as imaging findings strongly suggested malignancy, warranting radical nephroureterectomy. Lymphadenectomy was not performed due to the absence of radiological or intraoperative evidence of lymph node involvement. The IHC staining confirmed the diagnosis of urothelial carcinoma with characteristic expression patterns.

Case four

A 55-year-old male with no known comorbidities presented with a dull, aching type of pain in the left loin and hematuria for one month. He had no history of taking any hormonal medications. A 10x10 cm ballotable mass was palpable in the left lumbar region. Tenderness was present. A hypoechoic lesion of 8.7x8.3 cm in the upper and mid pole of the left kidney with pelvicalyceal system dilatation was seen on ultrasound.

**Figure 10:**
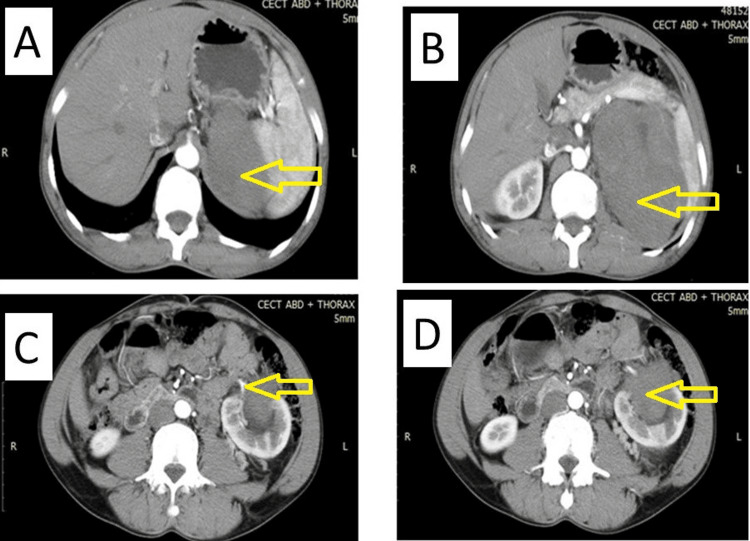
(A) A CT image showing a tumor arising from the left kidney; (B) A tumor displacing the stomach anteriorly, spleen anterolaterally with maintained fat planes; (C) Feeding artery noted arising from the left renal artery; (D) Exophytic mass protruding out from the renal pelvis CT: computed tomograhy

A large, well-defined heterogeneously enhancing mass (Figure 10) of 13x9.3x11.5 cm (anteroposterior (AP) x transverse (TR) x craniocaudal (CC)) was noted arising from the upper pole of the left kidney lesion and showed multiple non-enhancing hypodense regions within suggestive of necrotic areas with areas of internal calcifications and hyperdense areas suggestive of hematoma. The enhancement of the lesion in the corticomedullary and nephrogram phases was 50 Hounsfield units (HU) and 80 HU and showed significant enhancement in the delayed phase (98 HU).

Mass had displaced the stomach anteriorly, the spleen anterolaterally, and the left suprarenal gland displaced medially, posteromedially reaching up to the left psoas, causing compression with maintained fat planes in all planes. Inferiorly, the lesion extended to the renal hilum and renal pelvis. Posterolaterally, it was abutting the posterior abdominal wall muscles. It did not cross the midline and was abutting the paraspinal soft tissue with maintained fat planes. The feeding artery of the lesion was arising from the left renal artery. The patient was suspected to have left renal cell carcinoma.

**Figure 11:**
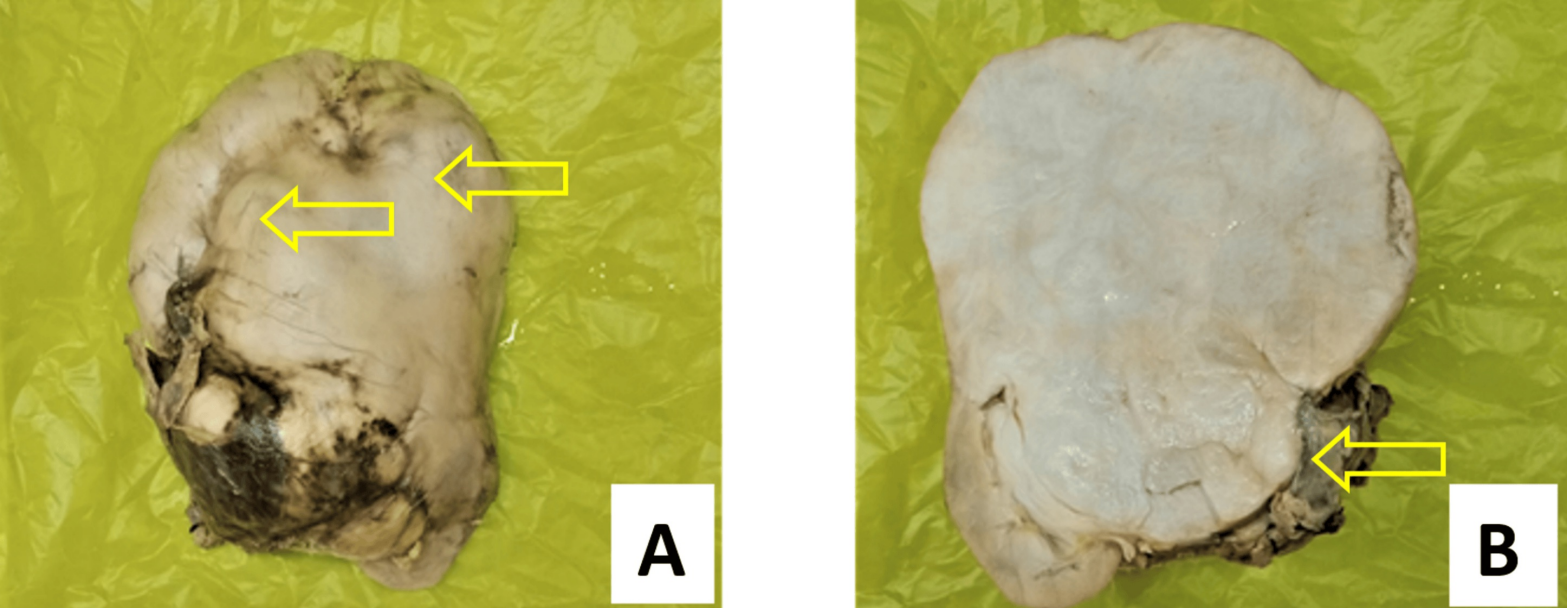
(A) Nodular and bosselated outer surface; (B) The cut surface having a well-circumscribed large gray-white solid tumor, displacing the kidney parenchyma into the periphery.

He underwent a left open radical nephrectomy. The specimen measured 16x13x6.5 cm (Figure 11). It had a nodular, enlarged, and bosselated outer surface. The cut surface had a large gray-white tumor measuring 12x10x3 cm with kidney parenchyma being displaced into the periphery. The tumor was well circumscribed and had gray-white solid areas. The renal pelvis at the inferior border was completely abutted by the tumor. The postoperative period was uneventful, and the patient has been doing well in the last six months with no complaints.

No biopsy was performed due to the large tumor size and clear imaging findings suggestive of renal cell carcinoma. Lymphadenectomy was not performed as imaging and intraoperative findings showed no evidence of lymph node involvement. The IHC staining report confirmed the diagnosis of renal cell carcinoma, with markers such as PAX8 and CD10 showing positive expression.

## Discussion

A MEST of the kidney is an uncommon benign tumor of the kidney distinguished histologically by a mixture of stromal and epithelial proliferation. Michal and Syrucek first proposed the term MEST in 1998 [4]. The tumor has cystic areas along with solid areas. Epithelial and stromal components of MEST are similar to adult cystic neoplasm (ACN) [5,6]. In the 2022 WHO classification of renal neoplasms, renal MEST along with ACN has been included [7].

Mixed epithelial and stromal tumors have a significant predisposition in middle-aged women taking estrogenic hormonal medications, inclining toward the possibility of hormonal stimulation resulting in the growth of these tumors [8]. The male-to-female ratio has been found to be 7:1 [7]. Pain, hematuria, or signs of urinary tract infection are the common symptoms. About 25% of lesions are picked up by imaging studies [9]. Radiologically, they have features of a pedunculated contrast-enhancing lesion with cystic, solid components encompassed between thickened irregular septae, similar to Type III or IV Bosniak cysts [10]. 

Unlike adult cystic neoplasm, the epithelial and stromal elements are complex in MEST. On histological examination, a biphasic pattern was observed, featuring columnar epithelial cells and spindle-shaped stromal components. These tumors are benign and usually do not show tissue invasion. However, to date, 18 borderline or malignant cases have been reported [11]. They tend to grow along the collecting system distally. As far as we are aware, this is the initial documented instance of the tumor extending from the renal pelvis through the entire course of the ureter extruding into the bladder.

The diagnosis of MEST was confirmed after the surgical procedure through pathological analysis, as no apt and reliable features have been established in preoperative imaging studies yet. Complete surgical resection is the modality of treatment either by partial or radical nephrectomy [12]. All efforts to attain a margin negative resection are necessary, as cases of recurrence have been reported [13]. The tumor should be handled with caution to avoid tumor spillage and prevent peritoneal seedling [14]. Although cases of malignant transformation have been noted, most MESTs have a benign course with favorable outcomes [10,15,16].

## Conclusions

To summarize, this study highlights the intraoperative, pathological, and immediate postoperative outcomes of laparoscopic resection for complex renal masses involving the collecting system. The findings in our case series emphasize the feasibility and effectiveness of complete surgical resection with negative margins in such cases. Further studies are needed to assess the long-term outcomes, including the potential risks of recurrence or malignant transformation.
